# Treatise on the Influenza of 1837

**Published:** 1838-01

**Authors:** 


ART. III.—*A Treatise on the Influenza of* 1837; *containing an Analysis of one hundred Cases observed at Birmingham.* By P. BLAKISTON, M.A. Med. Lic. Cantab., Physician to the Birmingham Dispensary.—*London,* 1837. 8vo. pp. 60.

THIS is the best treatise that we have yet seen on the subject of last year's epidemic. "It lays no claim to be considered as a history of the disease; . . . no attempt has been made to institute a comparison between this complaint and the epidemic of former years; . . . the author has confined himself to the simple task of recording *facts,* which he has endeavoured to observe accurately and to report faithfully; deducing such conclusions alone as appear incontestibly to result from their analysis." (*Pref.*) We think that Dr. Blakiston has fulfilled his promises, and attained his object; and his work will be useful to the future historian of the epidemic.

The following conclusions are regarded by the author as resulting from his investigations:

"1. The influenza, as observed at Birmingham, is an affection of the nervous system, with its concomitant derangements in the organs of digestion, circulation, &c. commonly known under the name of Nervous Fever; accompanied, throughout its whole course, by irritation of the pulmonary mucous membrane.

"2. This irritation not unfrequently amounted to congestion, and even to inflammation.

"3. The influenza was modified by pre-existing disease, more particularly by chronic bronchitis, the subjects of which were rendered liable to the acute form of that disease.

"4. Neither locality, previous habits, or diet, acted as predisposing causes.

"5. In simple, uncomplicated cases, mild treatment alone was sufficient.

"6. When bronchitis was present, counter-irritation, and large doses of ætherial tincture of lobelia, repeated at short intervals, seemed useful.

"7. Venesection was always counter-indicated.

"8. It was often necessary to have recourse to diffusible stimulants at the commencement of the complaint, and to administer tonic medicines in an early stage of it.

"9. It only proved fatal in those cases where the persons it attacked had been enfeebled by old age or chronic disease." (P. 59.)

